# Targeting c-Met in the treatment of urologic neoplasms: Current status and challenges

**DOI:** 10.3389/fonc.2023.1071030

**Published:** 2023-03-07

**Authors:** Pengxiao Su, Ming Zhang, Xin Kang

**Affiliations:** Honghui Hospital, Xi’an Jiaotong University, Xi’an, China

**Keywords:** c-Met, HGF, urologic neoplasms, renal cell carcinoma, prostate cancer, bladder cancer, tyrosine kinase inhibitors, CAR-T

## Abstract

At present, studies have found that c-Met is mainly involved in epithelial-mesenchymal transition (EMT) of tumor tissues in urologic neoplasms. Hepatocyte growth factor (HGF) combined with c-Met promotes the mitosis of tumor cells, and then induces motility, angiogenesis, migration, invasion and drug resistance. Therefore, c-Met targeting therapy may have great potential in urologic neoplasms. Many strategies targeting c-Met have been widely used in the study of urologic neoplasms. Although the use of targeting c-Met therapy has a strong biological basis for the treatment of urologic neoplasms, the results of current clinical trials have not yielded significant results. To promote the application of c-Met targeting drugs in the clinical treatment of urologic neoplasms, it is very important to study the detailed mechanism of c-Met in urologic neoplasms and innovate c-Met targeted drugs. This paper firstly discussed the value of c-Met targeted therapy in urologic neoplasms, then summarized the related research progress, and finally explored the potential targets related to the HGF/c-Met signaling pathway. It may provide a new concept for the treatment of middle and late urologic neoplasms.

## Introduction

Renal cell carcinoma (RCC), prostate cancer (PCa) and bladder cancer (BCa) are the most common urologic neoplasms, which are a major tumor system threatening human health. RCC is the 12th most common cancer worldwide (1). The most common histological subtype is renal clear cell carcinoma(RCCC) (2). PCa is the second most common cancer and the fifth leading cause of cancer death in men. In recent years, the incidence of PCa is increasing year by year (3). BCa is the tenth most common cancer worldwide. It is more common in men than in women, with morbidity and mortality rates of 9.5 and 3.3 per 100,000 population, respectively. As a result, the disease is more prevalent in men, for whom it is the sixth most common cancer and the ninth leading cause of cancer death (3). Therefore, due to the high incidence of urinary tract tumors, which seriously affects human health, a large part of the world’s medical and health resources should be used for the prevention and treatment of urologic neoplasms.

In recent years, with the promotion of early screening, early detection of tumors and the development, application of tumor diagnostic markers, the diagnosis and treatment of urologic neoplasms have made rapid progress (4–6). Because the progression of urologic neoplasms is slower than that of other systems, it is easy to achieve radical curative effect when tumors are detected at an early stage. However, due to the unbalanced development of economic level, early cancer screening and early detection cannot be quickly and comprehensively popularized, which leads to a large number of patients with middle and advanced urologic neoplasms are still found in clinical practice. For these patients, recurrence and metastasis after surgery are the focus of treatment. Therefore, it is pressing to exploit new targeted therapies for the treatment of middle and advanced urologic neoplasms.

C-Met, as a tyrosine kinase receptor, is overexpressed in multiple tumors and exerts an active function in tumor progression as an oncogenic factor (7). Because c-Met activation occurs in combination with HGF or through ligand-independent mechanisms (8), c-Met is often dysregulated in solid tumors, including urologic neoplasms (9–11). It is significantly overexpressed in tumor metastasis sites due to its properties of promoting tumor proliferation, angiogenesis and metastasis (12). Besides, c-Met was found to mediate the resistance signaling axis of single-dose immunotherapies targeting PD-1 (13). This observation provides a theoretical basis for the combined anti-tumor effect of immune checkpoint inhibitors (ICIs) and c-Met inhibition. Furthermore, c-Met itself can act as a tumor specific antigen and can be used as a precise guidance for T cells to eliminate tumor cells in immunotherapy. Therefore, targeting c-Met has great potential in the treatment of urologic neoplasms. In this review, we reviewed the expression of c-Met in urologic neoplasms tissues and its clinical prognostic value. Then, the mechanism of c-Met in urologic neoplasms and the studies of c-Met targeted therapy in urologic neoplasms were summarized. Finally, the potential therapeutic targets related to HGF/c-Met signaling pathway were discussed.

## Expression of c-Met in urologic neoplasms and its correlation with prognosis

Previous studies have demonstrated that c-Met overexpression exists in hepatobiliary tumors, so the treatment targeting c-Met has been carried out more frequently in the treatment of hepatobiliary tumors (14–16). However, recent studies have revealed that c-Met is also highly expressed in urologic neoplasms and is related with poor prognosis, indicating that c-Met is a potential target for urologic neoplasms.

## Renal cell carcinoma

In adult kidney, c-Met is expressed in renal tubular epithelial cells, and its main physiological function is to stimulate the growth of renal tubular cells (17, 18). Proper c-Met function is also crucial for inducing branching tubulogenesis during tubule repair after ischemia injury (19). Meanwhile, it has been shown that c-Met is involved in the progression of RCC as a proto-oncogene (20, 21).

Numerous studies have demonstrated that c-Met is overexpressed in RCC tissues and is closely related to pathological grade, stage and prognostic survival. It may have potential for prognostic assessment and targeted therapy (22–24). J. H. Kim et al. (25) conducted a study to evaluate the correlation between high c-Met expression and clinicopathologic factors and its impact on prognosis in RCC patients. Twelve studies involving 1724 patients were included. The results showed that compared with RCC with low c-Met expression, the tumor nuclear grade and pT stage were significantly higher with high c-Met expression. Besides, RCC patients with high c-Met levels had significantly lower overall survival (OS) than patients with low c-Met levels tumors. S. Macher-Goeppinger et al. (26) detected the expression of MET and the frequency of increased MET gene copies from the long-term follow-up data of patients with RCC based on a large hospital. The results showed that in 572 cases of RCC, 32% had high protein expression. High MET expression and increased MET copy number were also found to be related with an adverse patient outcome. These studies suggest that c-Met overexpression is present in RCC and overexpression is associated with significant malignant pathological features and poor survival. It also demonstrates that c-Met is a potential target for RCC treatment.

In addition, the expression of c-Met in chromophobe renal cell carcinoma (CRCC) and its prognostic significance have also been studied. F. Erlmeier et al. (27) evaluated the prevalence, distribution and prognostic impact of c-Met expression in CRCC. High expression of c-Met was found in 29.6% of patients, and there was a correlation between high expression of c-Met and lymph node metastasis. This suggests that the role and expression of c-Met in RCC progression may be universal and not limited by pathological types. This viewpoint also lays a theoretical foundation for the application of c-Met targeting in RCC.

## Prostate cancer

C-Met expression can be detected in normal prostate basal epithelial cells, but it is generally not expressed in peripheral and transitional zone epithelial cells (28). C-Met protein overexpression was found in 84% of primary PCa and 100% of metastatic PCa (29). These studies indicate that c-Met overexpression is significantly related to high-grade adenocarcinoma and may exert a crucial function in tumor progression. Besides, K. Nakashiro et al. (30)demonstrated that HGF produced by prostate-derived stromal cells stimulated the growth of androgen-dependent PCa cells *in vitro* and *in vivo*. It was found that epithelial cells began to express c-Met protein with the development of tumor malignancy. Therefore, the study have shown that the stromal cells of PCa may form an autocrine c-Met loop, which may act together with HGF expressed by cancer cells to promote tumor progression.

To further explore the role of c-Met in the prognosis of PCa, D. Strohmeyer et al. (31) demonstrated that the expression of vascular endothelial growth factor(VEGF) and c-Met increased with the increase of tumor stage and grade. In addition to VEGF, c-Met exerts a significant function in the induction of angiogenesis in PCa and is related to clinical prognosis. Furthermore, S. Nishida et al. (32) also found that the expression of HGF in prostate tissues was correlated with the biochemical recurrence of PCa after surgery. The results showed that patients with HGF overexpression in PCa had significantly longer biochemical relapse-free survival. Survival risk analysis showed that HGF overexpression was an independent risk factor for postoperative biochemical recurrence.

In addition, other researchers have investigated the correlation between c-Met protein expression and Gleason grade. F. Jacobsen et al. (33) successfully examined the expression of c-Met in 3378 PCa tissues by immunohistochemistry and analyzed the follow-up data of patients. The results demonstrated that c-Met protein was often overexpressed in PCa, and the high expression of c-Met protein was significantly correlated with high Gleason grade. These results indicate that c-Met is not only expressed in PCa tissues, but also involved in tumor progression. It is enough to confirm that c-Met is a meaningful target for PCa treatment. The above points also indicate that targeting c-Met in the treatment of PCa is theoretically feasible.

## Bladder cancer

As early as the 1990s, a study confirmed that the HGF/c-Met pathway was involved in the progression of BCa in animal models (34). Since then, the researchers have also compared HGF levels in the urine of BCa patients and healthy people, and found that HGF levels were significantly higher in BCa patients. Studies have shown that there seems to be a positive correlation between BCa progression and HGF levels (35). From then on, researchers have gradually begun to investigate the mechanism of HGF/c-Met signaling pathway in the progression of BCa.

K. Yamasaki et al. (36) retrospectively analyzed the expression of c-Met in tumor specimens of patients with invasive BCa and its relationship with prognosis. The results demonstrated that c-Met was highly expressed in 46% of cancer tissues. The overexpression of c-Met is significantly correlated with poor clinical prognosis, and the overexpression of c-Met indicates poor prognosis. Besides, X. Xu et al. (37) conducted a study to assess the pathological and prognostic role of c-Met status in BCa patients. Eight studies were eventually included, including 1,336 cases of BCa. The results showed that overexpression of c-Met in primary BCa was related to poor OS and was an independent risk factor for prognosis and survival. These studies suggest that c-Met is also involved in the progression of BCa and may be involved in the metastasis.

In addition, the correlation between c-Met and programmed death ligand 1 (PD-L1) in tumor tissues was investigated. Y. Mukae et al. (38) demonstrated that the high expression of c-Met was correlated with muscle invasion and metastasis of BCa, and c-Met exerted a vital function in invasion of tumor cell by regulating PD-L1. This study shows that c-Met is indeed involved in the invasion and metastasis of BCa, which again theoretically confirms that c-Met may affect the prognosis and survival of BCa patients. The above studies related to pathology and clinical prognosis also indicate that targeting c-Met may have great potential in the treatment of BCa.

## The mechanism of HGF/c-Met signaling pathway in urologic neoplasms

**Figure 1:**
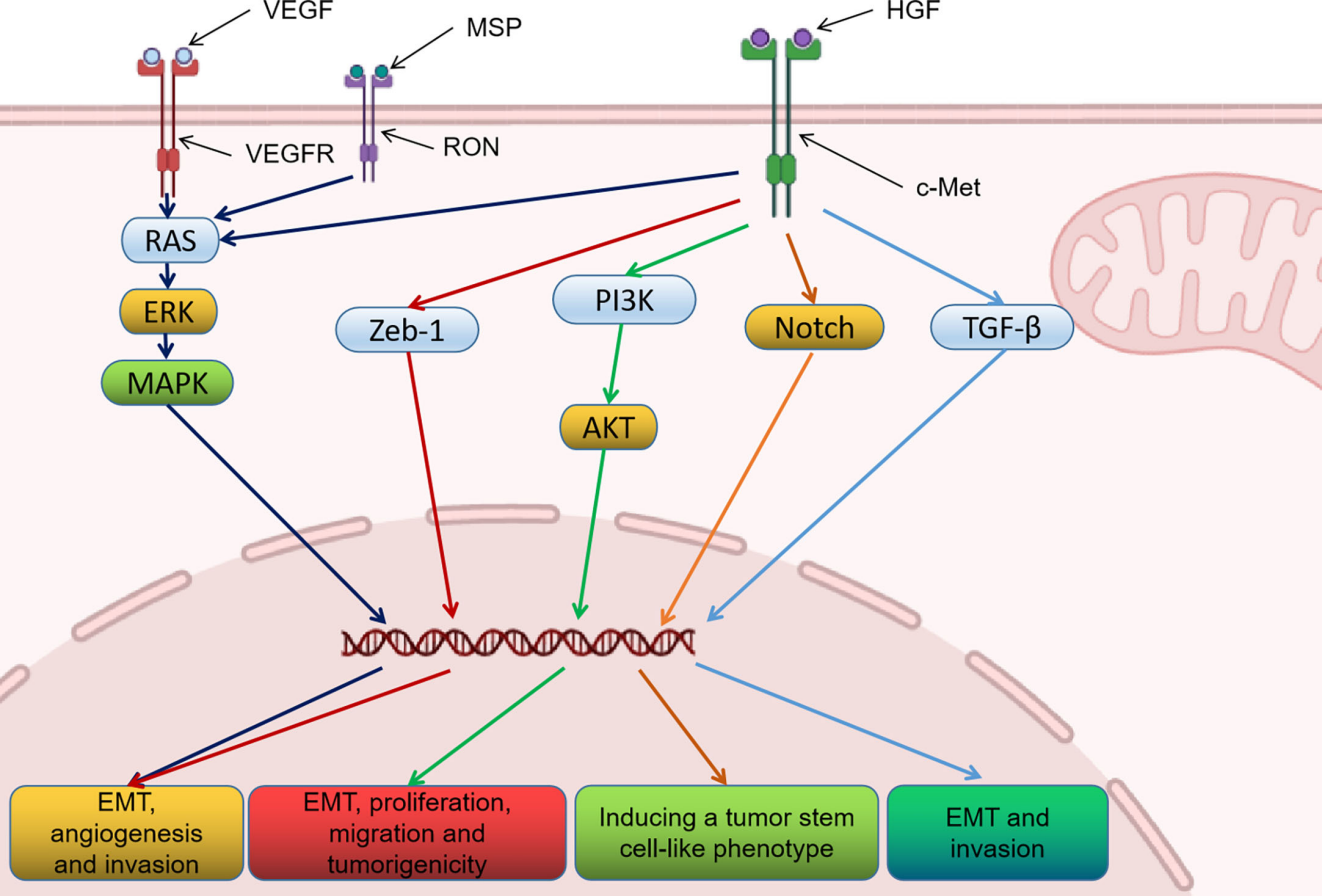
The role of c-Met signaling pathway in urinary tumor progression.

C-Met is a transmembrane tyrosine kinase receptor that is activated by HGF to regulate the expression of related downstream genes. This process is essential for cell migration under normal and pathological conditions. Current studies have demonstrated that c-Met is mainly involved in EMT in many types of cancer. HGF combined with c-Met promotes the mitosis of various tumor cells, and then induces motility, angiogenesis, migration and invasion. In recent years, many studies have been conducted on the oncogenic mechanism of c-Met in urologic neoplasms (**Table 1**). Studies have shown that c-Met is also involved in the formation of various phenotypes of urologic neoplasms through relevant signaling pathways (**Figure 1**). It is also confirmed that c-Met is a prospective therapeutic target for urologic neoplasms from the perspective of basic biology.

**Table 1:** Activation of HGF/c-Met signal in urologic neoplasms and related phenotypes.

Neoplasms	Mechanism	Phenotype	Reference
RCC	c-Met regulates VEGF expression	Inducing angiogenesis	A. Matsumura et al. (39)
PCa	RON and c-Met promote tumor metastasis through ERK signaling pathway.	Promoting tumor metastasis	B. Yin et al. (40)
PCa	ERK/MAPK and Zeb−1 signaling pathways	Increasing the invasive potential of PCa cells	Y. Han et al. (41)
PCa	The PI3K and MAPK signaling pathways	Enhancing cell proliferation, migration and tumorigenicity	Y. Han et al. (42)
PCa	Activation of stem cell-related Notch pathway	Inducing tumor stem cell-like phenotype	G. J. van Leenders et al. (43)
PCa	Inducing of intracellular reactive oxygen species, leading to the accumulation of DNA damage	c-Met signaling is used for survival and growth under androgen depletion conditions	A. Maeda et al. (44)
PCa	Increasing MMP-1, MMP-9, MT1-MMP, u-PA and uPAR in tumor cells	Increasing the invasive potential of PCa cells	Y. Fujiuchi et al. (45)
PCa	E-cadherin/catenin compounds	Influencing or regulating the adhesion between PCa cells	G. Davies et al. (46)
BCa	TGF-βsignaling pathway	Mediating tumor EMT and invasion	W. J. Sim et al. (47)

At present, numerous studies have demonstrated that HGF can regulate the expression of VEGF and promote tumor angiogenesis through its receptor c-Met (39, 48, 49). Researchers found that Von Hippel-Lindau (VHL) mutation and hypoxia resulted in increased expression of HGF and c-Met in RCC (50, 51). Besides, hypoxia-inducible factor 1 (HIF-1) during hypoxia can regulate the expression of c-Met and VEGF (52). Therefore, c-Met is a crucial target for anti-tumor angiogenic therapy of RCC.

In recent years, researchers have conducted some studies on the specific mechanism of EMT in PCa. B. Yin et al. (40) confirmed that HGF/c-Met signaling pathway may be the main mechanism of EMT in PCa. This study also found that RON and c-Met promote tumor metastasis through ERK signaling pathway. Besides, Y. Han et al. (41) demonstrated that HGF could induce tumor EMT by activating ERK/MAPK and Zeb-1 signaling pathways, thereby increasing the invasion potential of PCa cells. They also investigated the role of c-Met in EMT of PCa (42). The results showed that c-Met enhanced the proliferation, migration and tumorigenicity of tumor cells by regulating E-cadherin/vimentin. These EMT translation is mediated through PI3K and MAPK signaling pathways. G. Davies et al. (46) also found that the correlation between E-cadherin/catenin and c-Met may regulate the adhesion between PCa cells. Further studies demonstrated that HGF enhanced the invasive potential of PCa cells by increasing the production of MMP-1, MMP-9, MT1-MMP, u-PA and uPAR (45). These studies indicated that HGF/c-Met signaling pathway enhanced tumor invasion and metastasis in the process of promoting tumor EMT.

PCa consists of secretory cells and immature cells. C-Met was found to be specifically expressed in immature prostate cells. G. J. van Leenders et al. (43) determined the role of immature cells in PCa by analyzing the HGF/c-Met pathway. The results of this study show that HGF induces a molecular signature associated with stem cells by upregulating the activation of the Notch pathway. The results indicate that c-Met activation in PCa cells can induce tumor stem cell-like phenotype, and c-Met may regulate tumor invasion in surrounding tissues through Notch pathway. Besides, changes in c-Met overexpression in PCa tissues were found to be associated with tumor-independent androgen progression. Activation of c-Met signaling may induce spontaneous mutations or genomic instability leading to tumor progression in an androgen-independent state (44). The above studies indicate that c-Met can compensate for the deficiency of androgen in the progression of PCa, so targeting c-Met has special significance in the treatment of PCa.

In addition, aberrant HGF/c-Met upregulation and activation were found to be frequently observed in BCa and correlated with cancer progression and invasion. W. J. Sim et al. (47) found that HGF stimulated TGF-β signaling through SMURF2 signal pathway, leading to enhanced stability of TGF-β receptor. Finally, upregulation of TGF-β pathway by HGF leads to tumor EMT and invasion. Therefore, the investigators found that the combination of TGF-β receptor inhibitors may be promising in the treatment of BCa.

## Preclinical studies of c-Met targeting therapy in urologic neoplasms

Due to the role of c-Met in promoting the progression of urological neoplasms, c-Met targeting therapy have great potential in the treatment of urological neoplasms. Researchers have conducted a large number of preclinical studies in recent years, and preliminary results have been achieved (**Table 2**). Studies have shown that c-Met mediated signaling pathway exerts an important function in the progression of RCC. As an alternative pathway of VEGF, HGF/c-Met is emerging as a vital role in tumor angiogenesis and resistance to anti-VEGF therapy. The efficacy of simultaneous targeting of VEGF and c-Met in the treatment of RCC has been evaluated (56). The results demonstrated that the combination of axitinib and crizotinib could significantly improve the antitumor effect and prolong the survival time of the tumorigenic model. Honokiol (HNK) is a small molecule with antitumor effects. The researchers found that HNK exerts antitumor activity by inhibiting the c-Met-Ras axis (68). Besides, the antitumor effect of the combination of rapamycin and HNK in the treatment of RCC was also evaluated (54). The results show that the combination therapy can significantly inhibit the growth of RCC, which has significant therapeutic potential for the prevention of cancer after renal transplantation. In addition, other researchers have developed naturally occurring c-Met inhibitors for anti-tumor trials. K. Golovine et al. (55) studied the tumor killing activity of piperlongumine (PL) and found that PL rapidly reduced c-Met protein and RNA levels in RCC cells through a ROS-dependent mechanism. Therefore, PL has great potential in the adjuvant therapy of advanced RCC.

**Table 2:** Preclinical studies of c-Met targeting therapy in urologic neoplasms.

Conditions	Interventions	Models	Results	Reference
Papillary RCC	Anti-c-Met CAR-T cells	Cell lines and mouse models	Anti-c-Met CAR-T cells significantly inhibited tumor growth.	J. I. Mori et al. (53)
RCC	Honokiol and Rapamycin	Cell lines and mouse models	The combination of rapamycin and honokiol can effectively down-regulate the phosphorylation of Akt induced by c-Met in RCC cells, significantly inhibit tumor cell proliferation.	A. Sabarwal et al. (54)
RCC	Piperlongumine	Cell lines	PL inhibits tumor progression by rapidly reducing c-Met protein and RNA levels.	K. Golovine et al. (55)
RCC	Axitinib and crizotinib	Mouse models	The combination of axitinib and crizotinib also significantly enhanced the antitumor efficacy.	E. Ciamporcero et al. (56)
PCa	foretinib (GSK1363089)	Cell lines	Fretinib inhibits PCa cell metastasis by promoting the reversal of EMT.	B. Yin, et al. (40)
PCa	2,3,5,6-Tetrahydrobenzo[d]thiazole Derivatives	Cell lines	It showed high tumor suppressive activity.	R. M. Mohareb et al. (57)
PCa	PHA665752/Olaparib	Cell lines	Combination therapy has a synergistic effect on the growth inhibition of PCa cell lines.	Z. Wang et al. (58)
PCa with bone metastasis	cabozantinib	Cell lines and mouse models	Cabozantinib inhibited the growth of intraosseous tumors, decreased tumor-induced osteolysis.	C. Lee et al. (59)
PCa	Heteronemin	Cell lines	Heteronemin can effectively antagonize HGF/c-Met/STAT3 activation and tumor proliferation in refractory PCa cells.	J. C. Wu et al. (60)
PCa with bone metastasis	Axitinib and crizotinib	Mouse models	The combination of axitinib and crizotinib can significantly inhibit the bone damage of tumor cells, thereby significantly reducing osteoblastic and osteolytic lesions.	J. Eswaraka et al. (61)
PCa	SU11274	Cell lines	The inhibition of c-Met by SU11274 can significantly inhibit the survival and proliferation of tumor cells and enhance their radiosensitivity.	H. Yu et al. (62)
PCa	BMS-777607	Cell lines	BMS-777607 treatment significantly inhibited the proliferation, clonality, migration and invasion of PCa cells.	Y. Dai et al. (63)
PCa	Evodiamine	Cell lines	By inhibiting the activation of c-Met/Src/STAT3 signaling axis, it can inhibit the survival, proliferation and angiogenesis of tumor cells.	S. T. Hwang et al. (64)
PCa	Quercetin	Cell lines	Quercetin can reverse doxorubicin resistance in PCa cells by down-regulating c-Met expression.	Y. Shu et al. (65)
PCa	Curcumin	Cell lines	Curcumin can reverse HGF-induced EMT.	H. J. Hu et al. (66)
PCa	HGF RabMAb	Mouse models	Anti-HGF RabMAb not only inhibited the growth of tumor cells, but also inhibited the HGF-dependent proliferation of tumor cells.	Y. Yu et al. (67)

For the past few years, chimeric antigen receptor T cells (CAR-T) therapy has revealed remarkable efficacy in cancer immunotherapy, especially in the treatment of B-cell malignancies. To apply this technique to RCC, J. I. Mori et al. (53) developed c-Met targeting CAR-T cells for the treatment of papillary renal cell carcinoma (PRCC) and studied the anti-tumor efficacy of CAR-T cells. The results showed that a large number of c-Met targeting CAR-T cells infiltrated into tumor tissues and significantly inhibited tumor growth. Besides, the antitumor efficacy of CAR-T cells was synergistically enhanced when combined with axitinib. Due to the specific expression of c-Met in renal cancer tissues, CAR-T cells therapy targeting c-Met in renal cancer is expected.

Studies have shown that hormone-independent PCa is highly resistant to most conventional therapies, including radiation therapy, which is a major obstacle in the treatment of such patients. H. Yu et al. (62) shown that the inhibition of c-Met by SU11274 could significantly inhibit the survival and proliferation of DU145 cells and enhance their radiosensitivity. The potential mechanism may include inhibition of c-Met signaling, damage of DNA repair function and enhancement of cell death. This study is the first to demonstrate the efficacy of combining c-Met inhibition with ionizing radiation in the treatment of hormone-independent PCa. In addition, c-Met was found to be abnormally activated in the absence of HGF in many solid tumors (69). Y. Dai et al. (63) studied the reaction of PC-3 cells against HGF neutralizing antibody or small molecule c-Met kinase inhibitor (BMS-777607). The findings suggest that targeting c-Met in the absence of functional HGF remains a viable therapeutic option to halt cancer progression. These studies suggest that the antitumor activity of tyrosine kinase inhibitors (TKIs) targeting c-Met against PCa can be independent of the presence of HGF.

In addition, cabozantinib has been found in preclinical studies to reduce PCa growth in bone and has been shown to inhibit osteoblast activity. C. Lee et al. (59) found that the use of cabozantinib *in vivo* could inhibit c-Met and Vascular Endothelial Growth Factor Receptor 2 (VEGFR2) in osteoblasts, thereby reducing the expression of RANKL and M-CSF, and was associated with tumor-induced reduction of osteolysis. Other researchers have found similar results, J. Eswaraka et al. (61) tested the efficacy of axitinib combined with crizotinib in the treatment of castration resistant prostate cancer (CRPC) with bone metastases in a mouse model. The results showed that combined inhibitions of c-Met and VEGFR were helpful in the treatment of CRPC with bone metastases. Furthermore, molecular signal complementarity between RON and c-Met has been found (70), and some scholars have studied the anti-tumor efficacy of simultaneously targeting RON and c-Met. B. Yin et al. (40)demonstrated that foretinib (GSK1363089) inhibited the metastasis of PCa cells and promoted the reversal of EMT of PCa cells through the inhibition of RON and c-Met. Therefore, Foretinib with its broad tyrosine kinase inhibitory activity may hold promise in the treatment of metastatic PCa.

At present, the study of c-Met monoclonal antibody in urologic neoplasms is less. Only Y. Yu et al. (67) have developed anti-HGF rabmab, which can both block HGF/c-Met interaction and inhibit c-Met phosphorylation. The study confirmed the efficacy and potency of anti-HGF RabMAb in a mouse model of tumor transplantation. These results suggest that monoclonal antibodies targeting HGF may be a new therapeutic approach for advanced PCa. In addition, researchers have also found that curcumin (66), Heteronemin (60), heterocyclic compound (57) and Evodiamine(EVO) (64) can inhibit the progression of PCa by inhibiting HGF/c-Met pathway signaling. Quercetin can reverse doxorubicin resistance in PCa cells by down-regulating c-Met expression (65). These drugs are potential strategies in the treatment of PCa, and the specific efficacy needs to be confirmed in future clinical studies.

## Clinical studies of c-Met targeting therapy in urologic neoplasms

It is well known that patients with advanced urologic neoplasms have few treatment options. While these treatments may slow the progression of the disease, none is a complete cure. Therefore, it is necessary to continue to investigate other treatments for advanced urologic neoplasms. TKIs have been widely studied as a therapeutic approach for a variety of malignant tumors. As shown in **Table 3**, numerous clinical studies have been carried out in the treatment of urologic neoplasms with TKIs, some of which have been completed and some of which are being recruited. Most of the research was conducted in the United States, indicating that American institutions contributed significantly to the research. Published studies have shown that most TKIs targeting c-Met have good tolerability and safety in the treatment of urologic neoplasms. However, studies have found that most of the multi-target c-Met TKIs and combined with multi-target therapy have a good clinical response rate and clinical prognosis.

**Table 3:** Clinical studies of c-Met targeting therapy in urologic neoplasms.

Conditions	Interventions	First Posted	Number Enrolled	Phase	NCT (Number)	State	Status
RCC	foretinib	June 30, 2006	74	Phase 2	NCT00726323	United States	Completed
Papillary Renal Cell Cancer	AZD6094	April 30, 2014	111	Phase 2	NCT02127710	United States	Completed
RCC	APL-101/Nivolumab	September 5, 2018	20	Phase 1 Phase 2	NCT03655613	Australia	Terminated
RCC	INC280/bevacizumab	September 22, 2015	65	Phase 1	NCT02386826	United States	Active, not recruiting
Papillary RCC	Savolitinib/durvalumab/sunitinib	October 28, 2021	220	Phase 3	NCT05043090	United States	Recruiting
Advanced RCC	Drug: APL-101 Oral Capsules	September 27, 2017	344	Phase 1 Phase 2	NCT03175224	United States	Recruiting
Recurrent RCC	Erlotinib/Tivantinib	August 20, 2012	55	Phase 2	NCT01688973	United States	Completed
Advanced Solid Tumors	Axitinib/crizotinib	February 26, 2014	50	Phase 1	NCT01999972	United States	Completed
Advanced RCC Metastatic RCC	Cabozantinib	May 14, 2018	445	Phase 2	NCT03428217	United States	Completed
RCC	Cabozantinib	June 2013	658	Phase 3	NCT01865747	United States	Completed
Renal Clear Cell Carcinoma Renal Papillary Cell Carcinoma	Savolitinib/MEDI4736/Tremelimumab	January 2017	181	Phase 2	NCT02819596	United Kingdom	Active, not recruiting
Solid Tumors	INC280	February 29, 2012	131	Phase 1	NCT01324479	United States	Completed
Advanced Solid Tumors	Treatment with ARQ 197 in combination with sorafenib	September 2009	87	Phase 1	NCT00827177	United States	Completed
CRPC	Crizotinib/Enzalutamide	August 2014	24	Phase 1	NCT02207504	United States	Completed
CRPC	Tivantinib	January 11, 2012	78	Phase 2	NCT01519414	United States	Completed
PCa	Savolitinib/Durvalumab/Tremelimumab	December 12, 2017	500	Phase 2	NCT03385655	Canada	Recruiting
Urinary Bladder Neoplasms Ureteral Neoplasms Urethral Neoplasms	Crizotinib	September 27, 2016	8	Phase 2	NCT02612194	United States	Terminated

All clinicaltrials can be downloaded from www.clinicaltrials.gov (accessed October 5, 2022).

Cabozantinib is a TKI that inhibits both VEGFR and c-Met. Therefore, the antiangiogenic properties of cabozantinib have led to its use as a second-line therapy for metastatic Renal Cell Carcinoma (mRCC) and as a first-line treatment option when ICIs are contraindicated (71). Cabozantinib has been evaluated for safety and activity. G. Procopio et al. (72) conducted a multicenter study of Cabozantinib in the treatment of mRCC patients in Italy. Only 5 patients (5.0%) stopped treatment due to adverse events. Partial responses were observed in 35 patients (36%), stable disease in 33 patients (34%), and progression in 28 patients (30%). The median progression-free survival (PFS) was 8.0 months. This study have shown that Cabozantinib exhibits acceptable tolerability and antitumor activity. Furthermore, K. J. Peltola et al. (73) evaluated the expression of c-Met in mRCC patients treated with sunitinib. The results showed that patients with low c-Met expression had longer PFS and OS. Survival risk analysis showed that high c-Met expression was an independent risk factor for adverse PFS. Studies have demonstrated that targeting c-Met can provide a survival benefit for patients with mRCC.

The progression of PCa requires androgen support, and this characteristic determines that the treatment of PCa should not be without anti-androgen therapy. Therefore, most of the clinical trials of TKIs targeting c-Met are combination therapy. A. Tripathi et al. (74) conducted a phase I trial of crizotinib and enzalutamide (androgen receptor antagonist) in the treatment of CRPC, with the main purpose of investigating its safety and pharmacokinetics. The study found that concurrent administration of enzalutamide and crizotinib resulted in a clinically significant reduction of systemic crizotinib exposure of 74%. Meanwhile, P. G. Corn et al. (75) evaluated cabozantinib combined with androgen deprivation therapy (ADT) for metastatic PCa. The results showed that cabotinib combined with ADT had better clinical activity in the treatment of metastatic PCa. In addition, Tivantinib has been found to be mildly toxic and improve PFS in patients with asymptomatic or minimally symptomatic mCRPC (76). The above studies indicate that TKIs combined with special treatment determined by the characteristics of the tumor itself may produce better efficacy, which also proves that TKIs is only suitable for adjuvant treatment of advanced tumors.

At present, olaparib has been clinically approved for the treatment of PCa, but cytotoxicity and DNA damage limit its clinical application. Z. Wang et al. (58) found that the combined inhibition of c-Met and poly ADP-ribose polymerase(PARP) had a synergistic effect on blocking the growth of PCa cell lines. When the two drugs were combined, tumor invasion and migration were prominently inhibited. This study suggests that targeting both c-Met and PARP may be a valuable strategy for PCa treatment.

## Potential therapeutic targets related to the HGF/c-Met signaling pathway in urologic neoplasms

**Figure 2:**
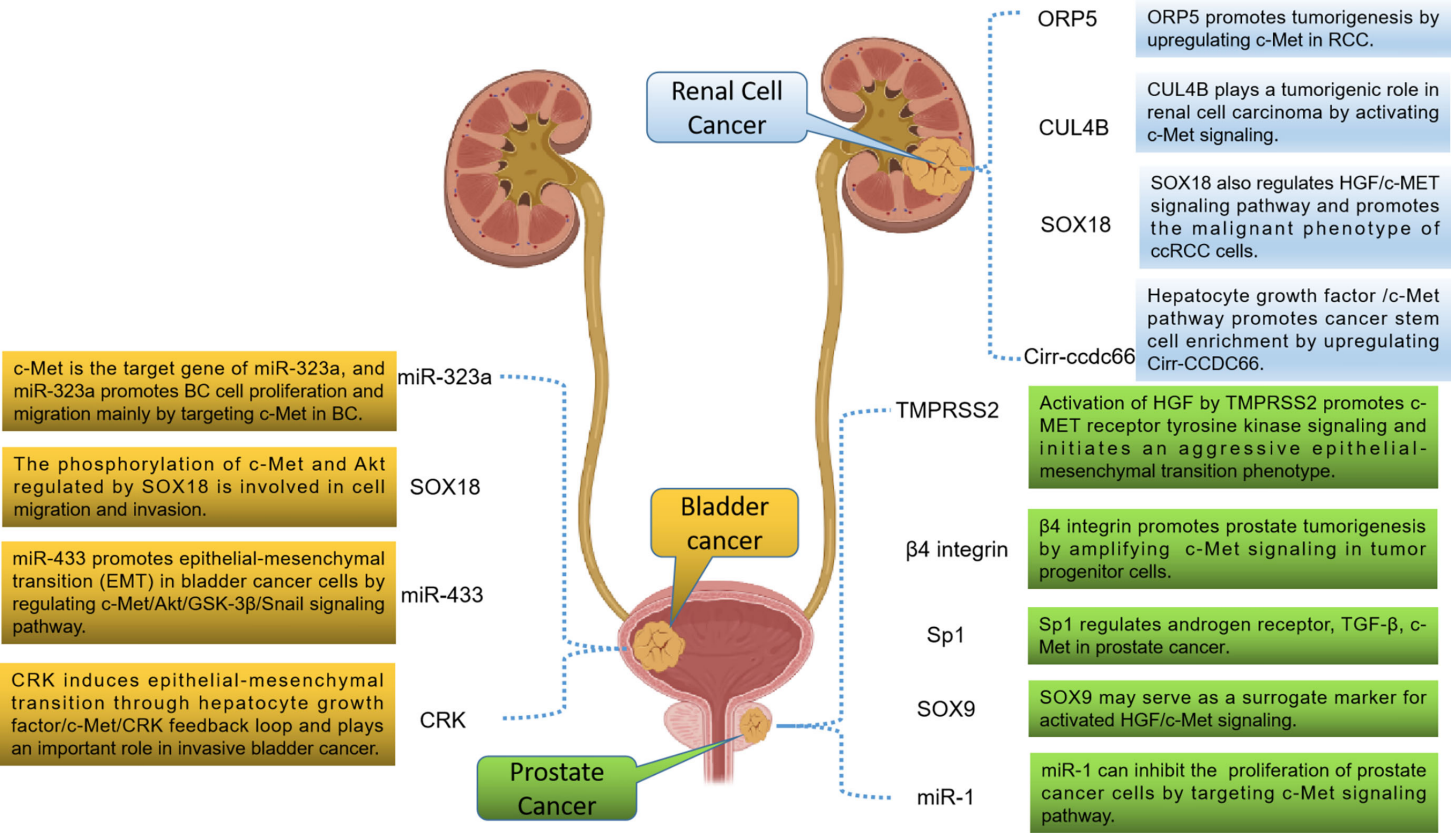
Potential therapeutic targets related to the HGF/c-Met signaling pathway in urologic neoplasms.

Previous studies have shown that HGF/c-Met signaling pathway exerts a crucial function in the progression of urologic neoplasms. Therefore, targeting HGF/c-Met signaling pathway is a hopeful approach for the treatment of urologic neoplasms. Besides, more and more studies have confirmed that HGF/c-Met signaling is also regulated by other targets. Some studies have also confirmed that tumor progression can be inhibited by inhibiting these targets. Therefore, targets that regulate the HGF/c-Met signaling pathway may also have potential value for targeted therapy (**Figure 2**).

Cullin 4B (CUL4B) is a structural protein encoding ubiquitin ligase complex, which is normally involved in physiological and developmental processes of the body. In recent years, it has been found that it is overexpressed as an oncogene in various solid tumors (77–79). S. Chen et al. (80) found that the expression of CUL4B in RCC cells and tissues was positively associated with the expression of c-Met. Further studies have demonstrated that CUL4B exerts a function in promoting tumor progression by activating c-Met signaling in RCC. Therefore, CUL4B may have potential value in the treatment of RCC. Besides, the transcription factor SOX18 has now been implicated in malignant tumor phenotype, angiogenesis, and lymphangiogenesis. Y. Huaqi et al. (81) found that activated SOX18 could induce HGF/c-Met signaling pathway both *in vitro* and *in vivo* in RCC. These results suggest that SOX18 may be a valuable target for the treatment of RCC. Previous studies have shown that circRNAs are involved in the occurrence and development of many cancers. J. Yang et al. (82) found that the HGF/c-Met pathway was activated during the enrichment of cancer stem cells and was responsible for the upregulation of circ-CCDC66. Inhibition of HGF/c-Met blocked circ-CCDC66-induced enrichment of cancer stem cells. It was confirmed that circ-CCDC66 may be also a therapeutic target for RCC. In addition, ORP5 is a lipid transporter that increases metastasis in a variety of cancers (83). L. Song et al. (84) also found that ORP5 promoted tumorigenesis by upregulating c-Met in RCC. These studies suggest that molecules that regulate HGF/c-Met signaling may be developed as therapeutic targets for RCC in the future.

TMPRSS2 is an androgen-regulated serine protease that has been found to be highly expressed in most metastatic PCa. J. M. Lucas et al. (85) found that TMPRSS2 initiated invasive EMT through activated HGF/c-Met signaling. The researchers also screened a potent TMPRSS2 inhibitor for *in vivo* studies and found that TMPRSS2 inhibitors inhibited PCa metastasis *in vivo*. Meanwhile, T. Yoshioka et al. (86) found that a large number of advanced PCa and CRPC expressed high levels of β4 integrin. Further studies revealed that β4 integrin is often co-expressed with c-Met and ErbB2 in PCa, and TKIs that simultaneously target these targets have shown significant ability to inhibit tumor progression in an *in vivo* model of PCa. These results suggest that β4 integrin, ErbB2 and c-Met may be involved in the occurrence and development of PCa through interaction. Besides, the specific protein (Sp) family has been shown to be involved in tumorigenesis. Studies have shown that Sp1 can regulate TGF-β, c-Met, PSA and α-integrin in PCa. These results indicate that Sp1 has potential value in targeted therapy for PCa because of its important role in PCa progression (87). Other researchers have investigated the mechanism of microRNA-1 (miR-1) in PCa cells (88). The results revealed that miR-1 promoted the proliferation of PCa cells by activating the c-Met/Akt/mTOR signaling pathway. Therefore, targeting MiR-1 may be used to treat PCa. In addition, H. Qin et al. (89) found that PCa cells regulate SOX9 molecules through the HGF/c-Met-ERK axis. SOX9 may serve as a surrogate marker for activated HGF/c-Met signaling to recruit optimal PCa patients for HGF/c-Met inhibitory therapy because it is more stable and easier to detect.

SOX18 is also a transcription factor that exerts a crucial function in regulating cell differentiation, lymphatic and vascular development. Y. Huaqi et al. (90) found that SOX18 promotes the migration and invasion of tumor cells by regulating c-Met and Akt, indicating that SOX18 plays a crucial role in BCa metastasis. There is growing evidence that dysregulation of certain microRNAs (miR) may contribute to tumor progression and metastasis. Xu X et al. (91) found that miR-409-3p was down-regulated in human bladder cancer tissues and cell lines. Overexpression of miR-409-3p in bladder cancer cells significantly reduced its migration and invasion. Further studies showed that miR-409-3p inhibited the expression of c-Met by binding to the 3 ‘ untranslated region of c-Met. These findings suggest that miR-409-3p may inhibit the progression of bladder cancer *via* the c-Met pathway. Meanwhile, J. Qiu et al. (92) studied and evaluated the expression and role of miR-323a in BCa progression. Studies have revealed that the reduced expression of miR-323a in BCa promotes the proliferation and migration of BCa cells mainly by targeting c-Met. Besides, X. Xu et al. (93) found that miR-433 promoted BCa EMT by regulating the c-Met/Akt/GSK-3β/Snail signaling pathway. Targeting miR-433 may be a new method to inhibit the progression and metastasis of BCa. In addition, CRK is an adaptor protein that plays a crucial role in the malignant potential of various invasive human cancers. R. Matsumoto et al. (94) demonstrated that CRK induced EMT through HGF/c-Met/CRK feedback loop in invasive BCa. Therefore, CRK may also be a significant target for BCa, especially in preventing metastasis.

## Discussion

This review have revealed that c-Met is highly expressed in urologic neoplasms and plays an significant role in tumor progression. Numerous studies have been conducted on c-Met targeted therapy in urologic neoplasms, and preclinical studies have shown obvious tumor suppressive activity. At present, published clinical studies have shown that most TKIs targeting c-Met have good tolerability and safety, and the combined multi-target treatment strategy has shown acceptable clinical response rate and prognostic survival in cancer treatment. Meanwhile, studies have also shown that TKIs targeting c-Met combined with VEGF, RON inhibitors or specific tumor treatment strategies (such as anti-androgen therapy for PCa) can achieve better clinical efficacy. Therefore, it also indicates that the current TKIs targeting c-Met are more suitable for adjuvant therapy of advanced urologic neoplasms.

C-Met interacts with other oncogenic molecules (such as EGFR and RON) to activate downstream pathways, thereby mediating tumor progression and drug resistance (69). Similarly, there is also signal interaction between c-Met and VEGF and RON molecules in urologic neoplasms (40, 56). This may also be a reason why c-Met targeting therapy has not achieved significant breakthroughs in the treatment of urologic neoplasms. Studies have also shown that targeting both c-Met and VEGF can achieve better tumor killing efficacy (59, 61). However, RON and VEGF are certainly not the only tumorigenic factors that interact with c-Met to promote tumor progression or generate drug resistance. Therefore, it is particularly crucial to study the mechanism of c-Met signaling pathway interaction in urologic neoplasms. Only by comprehensively mastering the signaling pathways and interaction mechanisms related to c-Met and tumor, we can better select the c-Met targeting therapy.

For the past few years, monoclonal antibodies against PD-1/PD-L1 and VEGF have been approved for clinical application in anti-tumor therapy. Besides, monoclonal antibodies against c-Met, such as Onartuzumab and Emibetuzumab, have been applied to digestive system tumors, and have shown good tolerance and clinical response rate (95, 96). However, there are few studies on these antibodies in urinary tract tumors. In the future, the monoclonal antibody against c-Met should be developed for the study of urologic neoplasms, or the targeted therapy of c-Met combined with the monoclonal antibody of PD-1/PD-L1 and VEGF should be used for the killing test of urologic neoplasms.

In addition, tumor killing by CAR-T cells technology depends only on the targeting of the target, not on the mechanism of the target. As a membrane protein specifically expressed in tumor cells, c-Met is suitable for tumor therapy using CAR T cell technology. CAR-T cells targeting c-Met have been studied in gastric cancer (97, 98), liver cancer (99, 100) and breast cancer (101), and good tumor killing activity has been achieved. Previous studies have demonstrated that c-Met plays a vital role in the progression of urologic neoplasms, and c-Met is highly expressed in tumor tissues. Therefore, CAR-T cells technology targeting c-Met has the potential for application in urologic neoplasms. However, only one c-Met-CAR-T cells study has been conducted in PRCC (53). In the future, c-Met-CAR-T cells technology should be widely studied in the treatment of urologic neoplasms to achieve good results.

## Conclusion

In conclusion, c-Met is involved in the progression of urologic neoplasms. It is highly expressed in tumor tissues and is associated with poor clinical prognosis. Due to the interaction mechanism between c-Met and other molecular signals, the use of targeting c-Met alone has limitations, while the combination of other antitumor methods showed better tumor killing efficacy. In the future, with the in-depth research on the mechanism of c-Met in urologic neoplasms and the optimization of CAR-T cells technology, it is believed that the targeted therapy of c-Met in the treatment of urologic neoplasms will surely make breakthrough progress.

## Author contributions

PS and XK also performed literature management and produced tables and graphs. All authors contributed to the article and approved the submitted version. 
